# Bone mineral density in very low birthweight adults—A sibling study

**DOI:** 10.1111/ppe.12876

**Published:** 2022-03-25

**Authors:** Samuel Sandboge, Juho Kuula, Johan Björkqvist, Petteri Hovi, Outi Mäkitie, Eero Kajantie

**Affiliations:** ^1^ Population Health Unit Finnish Institute for Health and Welfare Helsinki and Oulu Finland; ^2^ 7840 Psychology/Welfare Sciences Faculty of Social Sciences University of Tampere Tampere Finland; ^3^ Department of Radiology Medical Imaging Center University of Helsinki and Helsinki University Hospital Helsinki Finland; ^4^ Pediatric Research Center Children's Hospital University of Helsinki and HUS Helsinki University Hospital Helsinki Finland; ^5^ Folkhälsan Research Center Institute of Genetics Helsinki Finland; ^6^ Research Program for Clinical and Molecular Metabolism Faculty of Medicine University of Helsinki Helsinki Finland; ^7^ Department of Molecular Medicine and Surgery Karolinska Institutet, and Clinical Genetics Karolinska University Hospital Stockholm Sweden; ^8^ PEDEGO Research Unit MRC Oulu Oulu University Hospital and University of Oulu Oulu Finland; ^9^ 8018 Department of Clinical and Molecular Medicine Norwegian University of Science and Technology Trondheim Norway

**Keywords:** BMC, BMD, bone mineral content, bone mineral density, sibling study, very low birthweight, VLBW, VLBW adult

## Abstract

**Background:**

Children and adults born very low birthweight (VLBW, <1500 g) at preterm gestations have lower bone mineral density (BMD) and/or bone mineral content (BMC) than those born at term, but causality remains unknown.

**Objectives:**

Our aim was to assess BMD and BMC in adults born at VLBW in a sibling comparison setting to account for shared genetic and environmental confounders.

**Methods:**

We conducted a cohort study of 77 adults born VLBW and 70 same‐sex term‐born siblings at mean age of 29 years. The primary outcome variables were BMD Z‐scores, and BMC, of the femoral neck, lumbar spine, and whole body, measured using dual‐energy X‐ray absorptiometry. We analysed data by linear mixed models.

**Results:**

The VLBW adults had a 0.25 (95% CI 0.02, 0.47) Z‐score unit lower femoral neck BMD, and 0.35 (95% CI 0.16, 0.54) grams lower femoral neck BMC than their term‐born siblings, after adjustment for sex, age, and maternal smoking. Additional adjustment for adult body size attenuated the results. Lumbar spine, and whole body BMC were also lower in the VLBW group.

**Conclusions:**

Individuals born at VLBW had lower BMC values at all three measurement sites, as well as lower femoral neck BMD Z‐scores, compared to term‐born siblings, partly explained by their smaller adult body size, but the differences were smaller than those reported previously with unrelated controls. This suggests that genetic or environmental confounders explain partly, but not exclusively, the association between preterm VLBW birth and adult bone mineralisation.

**Synopsis**

1 **Study question**

Do adults born preterm at very low birthweight have lower bone mineral density or content than those born at term? Is this association explained by unmeasured shared familial confounders?

2 **What is already known**

Previous research suggests that children, adolescents, and adults born preterm have lower bone mineral density than term controls. To what degree this is explained by familial factors or environmental confounders, is unknown.

3 **What this study adds**

Preterm very low birthweight adults have lower bone mineral density and bone mineral content than their term siblings. The difference is smaller than in previous studies with unrelated controls suggesting that unmeasured confounders shared within family may explain a part of the difference.

## 1 BACKGROUND

Preterm birth is associated with an increased risk of adverse health outcomes, both in the short and long term. This association is more pronounced among those born preterm with very low birthweight (VLBW, <1500 g), the severity of health outcomes increasing in concordance with the degree of prematurity.^1^ The third trimester is critical for foetal skeletal development as most of the placental transport of calcium and phosphorous^2^ and up to 80% of foetal bone mineralisation occur during this period.^3^ Nearly one third of children born at VLBW suffer from metabolic bone disease of prematurity, a condition defined by radiological and biochemical signs of demineralisation.^2^, ^4^, ^5^, ^6^ Previous studies have not clearly established whether preterm birth is associated with long‐term detrimental effects on bone health.^2^ Comparisons of adolescents or young adults born preterm at VLBW with controls born at term generally report a lower bone mineral density (BMD) and/or bone mineral content (BMC) in those born preterm at VLBW^7^, ^8^, ^9^, ^10^, ^11^ while studies with different designs have not observed any such differences.^12^, ^13^, ^14^ At least a part of the reported difference is explained by smaller body size of those born preterm at VLBW.^7^, ^8^, ^9^, ^11^ The remaining difference could be a consequence of prenatal and neonatal events associated with preterm birth, or due to genetic and environmental characteristics associated with preterm birth and bone mineralisation. This has not been systematically studied.

To obtain more information regarding long‐term skeletal consequences of preterm birth, we compared bone health parameters in young adults born at VLBW with their same‐sex, term‐born siblings. This study design offers an opportunity to account for shared genetic and/or lifestyle factors regarding the association between preterm birth and later bone health.

## 2 METHODS

### 2.1 Cases and controls

The original study population consisted of 79 adults born at VLBW and 79 same‐sex siblings born at term, with less than a 10‐year age difference. The sibling pairs were recruited during 2014–2017 from the Helsinki Study of Very Low Birthweight Adults (HeSVA; *n* = 22),^15^ the Adults born Preterm in Northern Finland (ESTER; *n* = 6) study,^16^ and the Finnish Medical Birth Register (FMBR; *n* = 51). After initial assessment, eleven individuals were excluded: one sibling pair due to both becoming pregnant after recruitment, one pair due to compliance issues, three controls declined further participation, and four controls were found to be born preterm upon chart review. This left us with 77 VLBW participants and 70 controls. The recruitment process is described in detail previously.^17^

As part of the present study, the VLBW young adults and their siblings underwent dual‐energy X‐ray absorptiometry (DXA), and measurement of height and weight at a mean age of 29 years. Perinatal data were available for the HeSVA and ESTER subjects from earlier cohort studies, and hospital and maternity clinic records provided the corresponding data for the remaining VLBW participants and all siblings. The collected data included maternal age, parity, maternal smoking during pregnancy, birthweight, and gestational age.

The participants completed a questionnaire detailing, among other things, parental and own education and occupation, smoking status, and personal health and family history. Five individuals did not complete this questionnaire, four of whom additionally did not undergo DXA examination. Regarding questions pertaining highest attained parental education, 10 categories were available, *1: Not completed; 2: Primary school; 3: Lower Secondary/Comprehensive; 4: Vocational; 5: Upper Secondary; 6: College; 7: Polytechnic; 8: Lower tertiary; 9: Higher tertiary; 10: Doctoral degree*. These were reclassified into three categories to be used as a proxy for socioeconomic status: Lower secondary, or less (categories 1–3), Secondary (categories 4–7), and Tertiary (categories 8–10). In cases of mismatch between siblings, the replies were cross referenced with descriptive responses for parental occupation and corrected accordingly.

The DXA examination (Hologic Discovery A) included measurement of lumbar spine (L1‐L4), femoral neck, and whole body BMD and BMC. BMD values were transformed to Z‐scores based on age‐ and sex‐specific reference data for the equipment. We considered a BMD Z‐score below –1.0 as decreased. Because bone size influences lumbar spine BMD, we also estimated volumetric density with bone mineral apparent density (BMAD), calculated as BMAD = BMC_L1–L4_/bone area_L1–L4_^1.5^.^18^ By pairing with siblings rather than unrelated controls, potential familial confounding was largely accounted for. All scans were visually inspected, under supervision of an experienced reader (OM), and no significant scoliosis, vertebral compressions in the lumbar region, or foreign bodies, which could lead to measurement errors, were identified.

### 2.2 Statistical analysis

Mixed model linear regression provided measures of effect sizes and 95% confidence intervals (CI). This approach permits unmatched subjects to remain in the analysis to improve accuracy. BMD Z‐scores and BMC for femoral neck, lumbar spine, and whole body, calculated lumbar spine BMAD, and femoral neck area, were compared between VLBW and sibling controls in a series of mixed model linear regressions: model 1 adjusted for sex and age at clinical examination as well as maternal smoking during pregnancy, model 2 further included BMI, and model 3 further adjusted for height. Two indicator variables were used for maternal smoking based on smoking, non‐smoking, and unknown status. All continuous variables used in the analyses were normally distributed.

A product term between sex and the dichotomous VLBW variable was calculated to test for potential interaction. Linear regression models of BMD Z‐score (femoral neck, lumbar spine, or whole body) as dependent variable, and with sex and VLBW status as independent variables, were compared to equivalent models with the additional sex*VLBW interaction term. No indication of a sex*VLBW‐status interaction was found and accordingly, all analyses were performed with sexes combined. SPSS was used for all statistical analyses (IBM SPSS Statistics for Windows, version 27).

### 2.3 Missing data

In total, 77 VLBW subjects and 70 sibling controls (including 70 complete sibling pairs) underwent DXA analysis. In two subjects the proximal femur DXA assessment deviated from the study protocol and the values were omitted from the final analysis. For eleven subjects (4 VLBW and 7 siblings), some parts of whole body regions remained outside the scan area due to large body size, and equipment‐software‐derived estimates were used. In a sensitivity analysis, exclusion of these eleven subjects did not influence the associations between the dichotomous VLBW/control variable and whole body BMD and BMC. Therefore, these individuals were retained in analyses.

### 2.4 Ethics approval

The study was approved by the ethics committee of the Hospital District of Helsinki and Uusimaa. Informed consent was provided by all participants.

## 3 RESULTS

The study participants were on average 29 years old. VLBW subjects were shorter and lighter than the sibling controls, whose heights were close to the Finnish median heights (men 180 cm, women 165 cm). The proportion of mothers who smoked during pregnancy was similar between groups, around 15.0%. As for parental education, all subjects had at least one parent who had completed at least a secondary education and 61.4% had at least one parent who had completed a tertiary education. Baseline characteristics of the participants are shown in Table 1.

**TABLE 1:** Baseline characteristics of study participants

	VLBW subjects, *n* = 77	Term sibling controls, *n* = 70	Sibling pairs, *n* = 70
Women/Men	41/36	36/34	
	Mean (SD)	Mean (SD)	Mean difference (95% CI)
Age (years)	29.5 (2.8)	29.2 (5.1)	0.3 (−0.7, 1.3)
Height (cm)	167.6 (9.4)	172.6 (9.5)	−4.3 (−6.2, −2.4)
Weight (kg)	69.1 (15.4)	73.9 (17.1)	−4.7 (−8.2, −1.2)
BMI (kg/m^2^)	24.5 (4.7)	24.6 (4.5)	−0.3 (−1.5, 0.9)
Gestational age (weeks)	29.5 (2.5)	39.8 (1.3)	−10.3 (−11.0, −9.7)
Birthweight (g)	1150 (220)	3400 (430)	−2240 (−2360, −2120)
Parental characteristics			
Maternal age at birth (y)	29.7 (4.9)	30.1 (5.1)	−0.3 (−1.3, 0.7)
Maternal smoking during pregnancy			
Smoking (*n*, %)	11 (14.3)	11 (15.7)	
Non‐smoking (*n*, %)	64 (83.1)	53 (75.7)	
Unknown (*n*, %)	2 (2.6)	6 (8.6)	
Completed educational level of at least one parent (%)			
Lower secondary or less	0		
Higher secondary	38.6		
Tertiary	61.4		

Note

Means and standard deviations for the VLBW and term sibling groups are presented together with mean differences with 95% confidence intervals.

**FIGURE 1:**
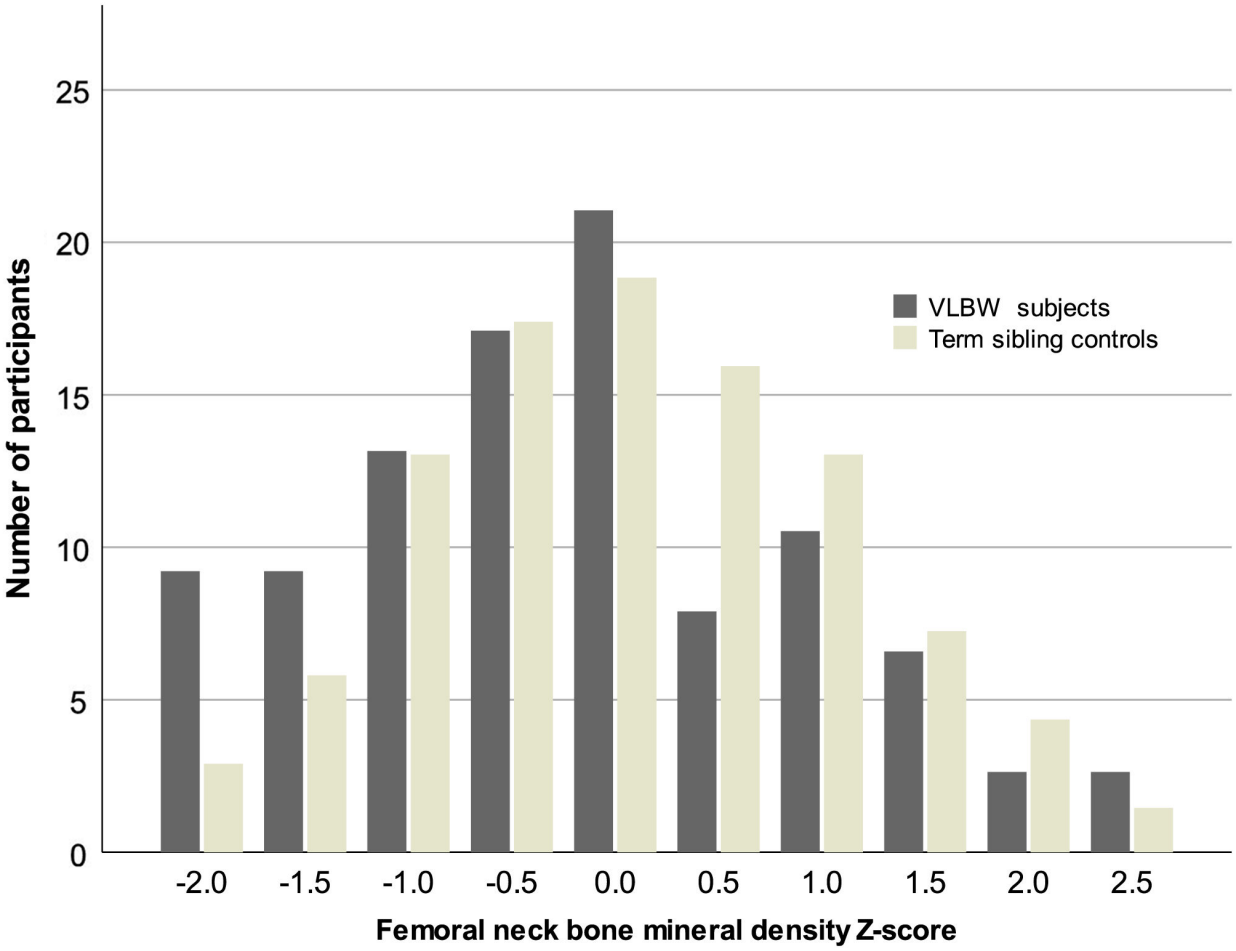
Femoral neck bone mineral density distributions in VLBW subjects and their term siblings. Mean femoral neck BMD Z‐score was lower in the VLBW (<1500 g) group (dark bars) than in the sibling group born at term (light bars). Individual Z‐score values are displayed within 0.5 Z‐score increments

Bone mineral content measurements were lower for all examined locations (femoral neck, lumbar spine, and whole body) in those born at VLBW, as compared to term‐born siblings (Table 2). Additionally, femoral neck BMD Z‐score (Figure 1) and femoral neck area were lower for those born at VLBW. No apparent differences between the VLBW subjects and siblings were seen for BMAD or BMD Z‐scores at lumbar spine or whole body.

**TABLE 2:** DXA assessment between VLBW and term sibling participants

	VLBW subjects, *n* = 77	Term sibling controls, *n* = 70	Sibling pairs *n* = 70
	Mean (SD)	Mean (SD)	Mean difference (95% CI)
Femur			
BMD femoral neck Z‐score	−0.2 (1.1)	0.1 (1.0)	−0.3 (−0.5, −0.05)
BMC femoral neck (g)	4.9 (1.1)	5.3 (1.0)	−0.4 (−0.6, −0.2)
Femoral neck area (cm^2^)	4.9 (0.5)	5.1 (0.5)	−0.2 (−0.3, −0.07)
Lumbar spine			
BMD L1‐L4 Z‐score	−0.04 (1.2)	0.01 (0.9)	−0.09 (−0.4, 0.2)
BMC L1‐L4 (g)	66.1 (15.3)	70.1 (14.2)	−3.9 (−6.7, −1.1)
BMAD L1‐L4 (g/cm^2^)	0.16 (0.02)	0.16 (0.02)	−0.001 (−0.005, 0.003)
Whole body			
Whole body BMD Z‐score	0.6 (1.0)	0.7 (1.0)	−0.09 (−0.3, 0.1)
Whole body BMC (g)	2682 (562)	2952 (646)	−244 (−340, −149)

Note

Means and standard deviations for the VLBW and term sibling groups are presented together with mean differences with 95% confidence intervals.

BMD, Bone mineral density g/cm^2^; BMC, Bone mineral content, g; BMAD, Bone mineral apparent density (Bone mineral content L1–L4 divided by area L1–L4^1.5^).

**FIGURE 2:**
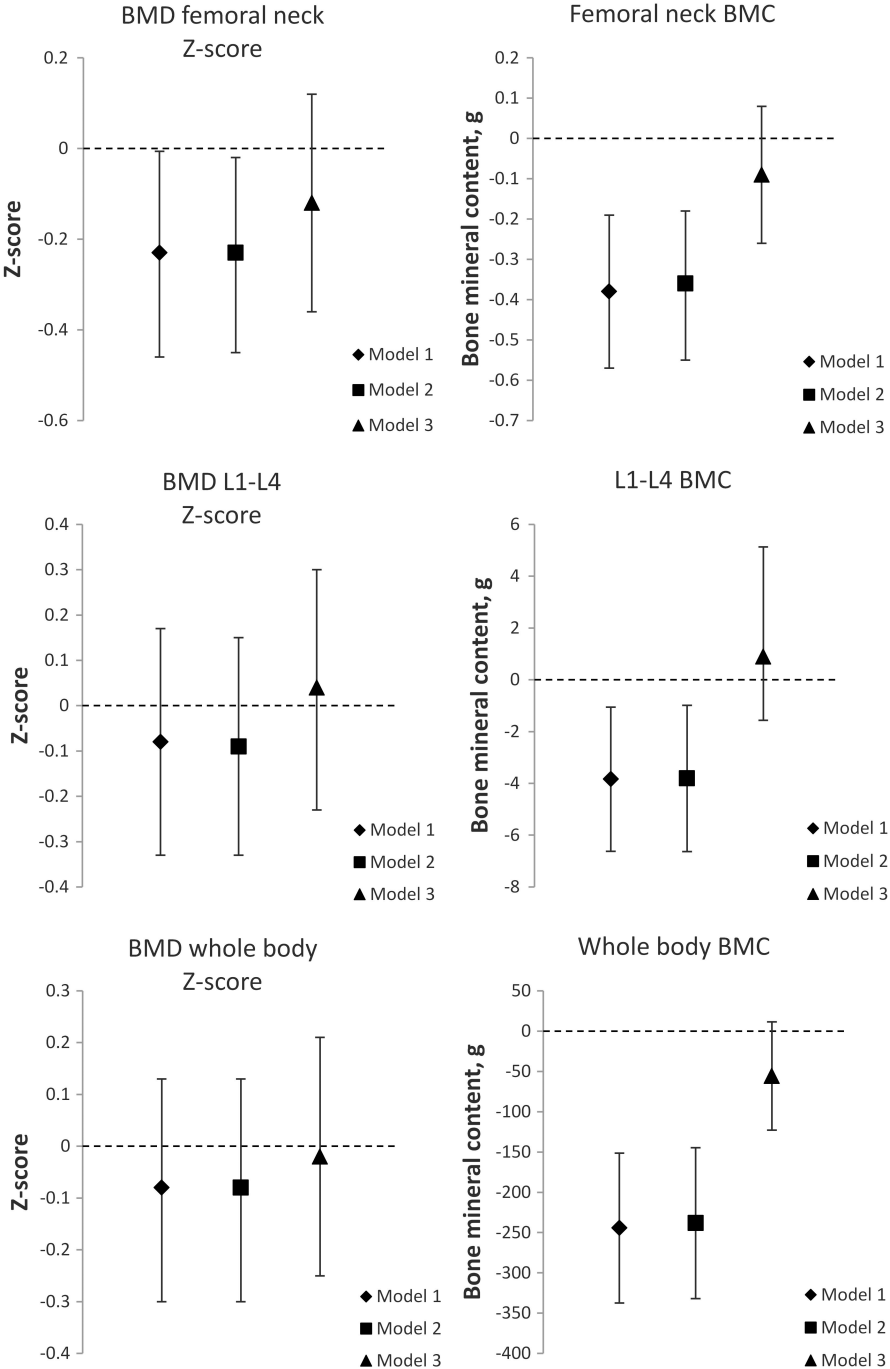
Mean differences (95% CI error bars) in bone mineral density Z‐scores and bone mineral content in femoral neck, lumbar vertebrae 1–4, and whole body (95% CIs, error bars) in adults born at VLBW compared to their term‐born siblings (zero line). Model 1 adjusted for maternal smoking during pregnancy, sex and age at examination, model 2 further adjusted for BMI, and model 3 further adjusted for height

After initial adjustment for maternal smoking during pregnancy, sex, and age (Table S1, model 1), femoral neck BMD Z‐score (−0.25, 95% CI −0.47, −0.02), and femoral neck area (−0.15 cm^2^, 95% CI −0.25, −0.06) were lower among VLBW subjects. In addition, BMC (g) at femoral neck (−0.35, 95% CI −0.54, −0.16), lumbar spine (−3.60, 95% CI −6.37, −0.82), and whole body (−236, 95% CI −332, −141) were lower among those born at VLBW compared to sibling controls (Table S1 and Figure 2). The differences were attenuated after further adjustment for height (Table S1, model 3). We did not adjust for attained parental education, due to its being identical in VLBW subjects and sibling controls. A larger proportion of the VLBW subjects had decreased (<−1.0) BMD Z‐scores at the level of the femoral neck (26.0% vs. 15.7%) and lumbar spine (23.4% vs. 15.7%). Among the individuals born at VLBW, three (3.9%) met the BMD criterion for osteoporosis (Z‐score below −2.5) at lumbar spine. No other BMD Z‐score measurements fell below the −2.5 threshold.

## 4 COMMENT

### 4.1 Principal findings

Our results suggest that adults born preterm with VLBW display lower bone mineral density and bone mineral content at almost thirty years of age, compared to their term‐born siblings. More specifically, the VLBW group presented lower Z‐scores at the femoral neck, with the difference surviving adjustment for maternal smoking during pregnancy, age, sex, and BMI, but attenuated when further adjusting for height. In addition, the BMC values at baseline were lower at all examined sites among the VLBW subjects but the differences also attenuated in the fully adjusted models. The differences were somewhat smaller than in previous studies comparing VLBW adults with unrelated controls. Our findings indicate that the lower DXA‐derived BMD and BMC values found in the subjects born preterm at VLBW are partly but not exclusively due to genetic or environmental factors shared within siblings. Moreover, these differences are to a substantial extent explained by the smaller body size of VLBW adults.

### 4.2 Strengths of the study

The study design has several strengths. Having siblings as controls adjusts for potential familial residual confounders and increases statistical power. Care was taken to collect perinatal records as accurately and completely as possible, and our study has only few unknown maternal data.

### 4.3 Limitations of the study

The sibling study design confers a risk of bias in overmatching, as behaviourally similar siblings might be more likely to participate. Our design also inherently excludes VLBW subjects without a suitable sibling, which may be a source of bias and decrease generalisability. We do not explore adverse bone health outcomes, and whether decreased BMC and BMD manifests as increased incidence of fractures remains to be seen.

### 4.4 Interpretation

It remains unestablished whether the lower BMD and BMC values *per se* could have adverse effects later in life. Preterm birth has been linked to an increased risk of fracture‐related hospitalisations with the association being strongest in early childhood.^19^ Preliminary epidemiological data suggest that, compared to individuals born at term, those born extremely preterm (<28 weeks) are less likely to suffer osteoporotic fractures during childhood and early adulthood.^20^ The shorter adult height among those born at VLBW could therefore be a protective factor, given the positive association between height and the risk of osteoporotic fractures.^21^, ^22^ Conversely, BMD seems to be an independent risk factor for fractures^23^ and whether the lower BMD in VLBW subjects might confer an elevated risk for fractures, with increasing age, is unknown. Furthermore, low BMI especially in males has been associated with a greater risk for falls^24^ and it remains unknown whether this applies to VLBW subjects.

The observed differences in BMD between VLBW and term‐born adults are smaller than those previously reported in the Helsinki Study of Very Low Birthweight Adults (overlap with current study was 22 VLBW participants). In that study, at mean age 23 years, the 144 VLBW participants had a 0.56 (95% CI −0.78, −0.34) unit lower femoral neck BMD Z‐score and a 0.51 (95% CI −0.75, −0.28) unit lower lumbar spine BMD Z‐score, compared to the 139 controls.^7^ These differences attenuated to 0.40 (95% CI −0.64, −0.17) and 0.26 (95% CI −0.51, −0.01), respectively, after adjustment for height, BMI, and exercise frequency. The Norwegian NTNU Low Birthweight Life study compared 52 VLBW subjects to 75 controls born at term at 25–28 years of age.^8^ In that study, BMD Z‐scores for the VLBW subjects were lower at the femoral neck (0.6 SD), total hip (0.4 SD), whole body (0.5 SD), and lumbar spine (0.3 SD). The differences for the first three locations remained after adjustment for height and weight.

A study from Montreal compared 101 18–29‐year‐olds born at <29 weeks with 95 friends or siblings recruited as controls.^9^ Femoral neck BMD Z‐score was 0.3 Z‐score units lower (95% CI −0.6, −0.0) among those born VLBW, after adjustment for height Z‐score, fat mass, and lean mass. Lumbar spine and whole body BMD Z‐scores were also lower in the VLBW group, but the differences were less pronounced, −0.2 units (95% CI −0.4, 0.1) for both locations.

These previous studies suggest that VLBW or very preterm born adults display lower BMD and BMC than their term‐born peers, which is in part explained by their smaller body size. The association was weaker in the present study, which used siblings as controls, and also in the Montreal study that used friends and siblings as controls. This suggests that the association is partly due to shared genetic or environmental factors within a family. Indeed, twin studies have demonstrated that features of skeletal size, and bone mass, are highly heritable.^25^

The lower baseline BMD among our VLBW subjects might still constitute a risk factor for future fractures, despite being primarily explained by a smaller body size. BMD is an independent risk factor for fractures; a 1 SD decrease in BMD is associated with a 1.5 to 3‐fold increase in fracture risk, and the strength of the association is similar, for example, to that between blood pressure and stroke.^23^ BMD also decreases with age and thus, individuals born at VLBW might be at higher risk of developing osteoporosis and fragility fractures earlier in life than term‐born individuals. Additionally, VLBW subjects have reported lower frequency of physical activity which might also contribute to an increased risk of osteoporosis during adulthood.^26^, ^27^ On the other hand, given the positive association between height and fracture risk, the relatively shorter stature among those born at VLBW might mitigate the association between BMD and osteoporotic fractures. Future follow‐up of the existing VLBW cohorts, the oldest of which including ours are currently in their 30s‐40s, will be required to confirm actual risk of osteoporotic fractures and inform preventive measures.

### 4.5 Conclusions

This study assesses the association between preterm birth and later bone health in a sibling setting. We found that individuals born at VLBW on average had lower femoral bone mineral density and bone mineral content, as well as lower bone mineral content in the lumbar spine, and whole body, than their term‐born siblings. These differences attenuated after adjustment for BMI and height indicating that the differences are primarily explained by the smaller body size of the VLBW subjects. Compared to previous studies on the association between preterm birth and later bone health, the differences in this study were of smaller magnitude. This could indicate that familial factors, such as shared genetic and/or lifestyle factors, have a substantial impact on later bone health, in addition to that of gestational length and/or intrauterine growth. The lower BMD among those born at VLBW might also constitute an independent risk factor for later fracture risk, especially considering the decline in BMD with increasing age, which might significantly impact later morbidity and mortality.

## CONFLICT OF INTEREST

The authors declare no competing interests.

## AUTHOR CONTRIBUTIONS

SS processed the data, performed the analyses, and was the primary author of the manuscript throughout the process, together with JK. JK was the primary agent of the sibling study, and was widely responsible for participant recruitment, logistics of the clinical study, and data collection and analysis. JB was instrumental in collecting and processing the data from patient records, and he reviewed and revised the manuscript. PH and OM contributed to the design and conduct of the sibling study and reviewed and revised the manuscript. EK, as a PI of the study, conceptualized and designed the study at large, provided funding, supervised data collection, and reviewed and revised the manuscript. All authors approved the final manuscript as submitted and met the ICMJE criteria for authorship.

## Supporting information

Table S1

## Data Availability

The datasets presented in this article cannot be made openly available due to legal and ethical reasons. The authors welcome requests for collaboration. Requests to access the datasets should be directed to the corresponding author or to kirjaamo@thl.fi. Requests may be subject to ethics approval and/or participant consent.

## References

[1] Raju TNK , Buist AS , Blaisdell CJ , Moxey‐Mims M , Saigal S . Adults born preterm: a review of general health and system‐specific outcomes. Acta Paediatr. 2017;106(9):1409‐1437. 28419544 10.1111/apa.13880

[2] Sharp M . Bone disease of prematurity. Early Hum Dev. 2007;83(10):653‐658. 17881164 10.1016/j.earlhumdev.2007.07.009

[3] Kovacs CS , Kronenberg H . Pregnancy and Lactation. 2018;147‐54.

[4] Nallagonda S , Nallagonda M , Deorukhkar A . Metabolic bone disease of prematurity – an overview. Paediatrics Child Health. 2016;27:14‐17.

[5] Done SL . Fetal and neonatal bone health: update on bone growth and manifestations in health and disease. Pediatr Radiol. 2012;42(Suppl 1):S158‐S176. 22395728 10.1007/s00247-011-2251-8

[6] Faienza MF , D'Amato E , Natale MP , et al. Metabolic bone disease of prematurity: diagnosis and management. Front Pediatr. 2019;7:143. 31032241 10.3389/fped.2019.00143 PMC6474071

[7] Hovi P , Andersson S , Jarvenpaa AL , et al. Decreased bone mineral density in adults born with very low birth weight: a cohort study. PLoS Medicine. 2009;6(8):e1000135. 19707270 10.1371/journal.pmed.1000135 PMC2722726

[8] Balasuriya CND , Evensen KAI , Mosti MP , et al. Peak bone mass and bone microarchitecture in adults born with low birth weight preterm or at term: a cohort study. J Clin Endocrinol Metab. 2017;102(7):2491‐2500. 28453635 10.1210/jc.2016-3827

[9] Xie LF , Alos N , Cloutier A , et al. The long‐term impact of very preterm birth on adult bone mineral density. Bone Rep. 2019;10:100189. 30627597 10.1016/j.bonr.2018.100189 PMC6319299

[10] Schlüssel MM , dos Santos VJ , Kac G . Birth weight and adult bone mass: a systematic literature review. Osteoporos Int. 2010;21(12):1981‐1991. 20419292 10.1007/s00198-010-1236-z

[11] Weiler HA , Yuen CK , Seshia MM . Growth and bone mineralization of young adults weighing less than 1500 g at birth. Early Hum Dev. 2002;67(1–2):101‐112. 11893441 10.1016/s0378-3782(02)00003-8

[12] Christmann V , van der Putten ME , Rodwell L , et al. Effect of early nutritional intake on long‐term growth and bone mineralization of former very low birth weight infants. Bone. 2018;108:89‐97. 29289790 10.1016/j.bone.2017.12.022

[13] Breukhoven PE , Leunissen RW , de Kort SW , Willemsen RH , Hokken‐Koelega AC . Preterm birth does not affect bone mineral density in young adults. Eur J Endocrinol. 2011;164(1):133‐138. 21030495 10.1530/EJE-10-0573

[14] Dalziel SR , Fenwick S , Cundy T , et al. Peak bone mass after exposure to antenatal betamethasone and prematurity: follow‐up of a randomized controlled trial. J Bone Miner Res. 2006;21(8):1175‐1186. 16869715 10.1359/jbmr.060516

[15] Hovi P , Andersson S , Eriksson JG , et al. Glucose regulation in young adults with very low birth weight. N Engl J Med. 2007;356(20):2053‐2063. 17507704 10.1056/NEJMoa067187

[16] Sipola‐Leppanen M , Vaarasmaki M , Tikanmaki M , et al. Cardiometabolic risk factors in young adults who were born preterm. Am J Epidemiol. 2015;181(11):861‐873. 25947956 10.1093/aje/kwu443 PMC4445394

[17] Björkqvist J , Kuula J , Kuula L , et al. Chronotype in very low birth weight adults ‐ a sibling study. Chronobiol Int. 2020;37(7):1023‐1033. 32354238 10.1080/07420528.2020.1754847

[18] Carter DR , Bouxsein ML , Marcus R . New approaches for interpreting projected bone densitometry data. J Bone Miner Res. 1992;7(2):137‐145. 1570758 10.1002/jbmr.5650070204

[19] Michaud J , Luu TM , LeBlanc JC , Healy‐Profitós J , Ayoub A , Auger N . Preterm birth and the future risk of orthopedic fracture. Pediatr Res. 2020;88(3):466‐472. 31968355 10.1038/s41390-020-0771-3

[20] Miettinen M , Alenius S , Nurhonen M , Salmi S , Nasanen‐Gilmore P , Haaramo P , et al. Preterm birth and risk of bone fractures during childhood and early adulthood. DoHAD; Melbourne2019. 10.1093/jbmr/zjaf011 PMC11909738 39843163

[21] Armstrong ME , Kirichek O , Cairns BJ , Green J , Reeves GK . Relationship of height to site‐specific fracture risk in postmenopausal women. J Bone Miner Res. 2016;31(4):725‐731. 26572496 10.1002/jbmr.2742 PMC4832288

[22] Xiao Z , Ren D , Feng W , Chen Y , Kan W , Xing D . Height and risk of hip fracture: a meta‐analysis of prospective cohort studies. Biomed Res Int. 2016;2016:2480693. 27818998 10.1155/2016/2480693 PMC5080474

[23] Compston JE , Cooper C , Kanis JA . Bone densitometry in clinical practice. BMJ. 1995;310(6993):1507‐1510. 7787600 10.1136/bmj.310.6993.1507 PMC2549881

[24] Yi SW , Kim YM , Won YJ , Kim SK , Kim SH . Association between body mass index and the risk of falls: a nationwide population‐based study. Osteoporos Int. 2021;32(6):1071‐1078. 33411009 10.1007/s00198-020-05725-1

[25] Peacock M , Turner CH , Econs MJ , Foroud T . Genetics of osteoporosis. Endocr Rev. 2002;23(3):303‐326. 12050122 10.1210/edrv.23.3.0464

[26] Kaseva N , Wehkalampi K , Strang‐Karlsson S , et al. Lower conditioning leisure‐time physical activity in young adults born preterm at very low birth weight. PLoS One. 2012;7(2):e32430. 22384247 10.1371/journal.pone.0032430 PMC3288099

[27] Tikanmaki M , Kaseva N , Tammelin T , et al. Leisure time physical activity in young adults born preterm. J Pediatr. 2017;189:135‐42.e2. 28751124 10.1016/j.jpeds.2017.06.068

