# Supplementary material for: Bone mineral density in very low birthweight adults—A sibling study
Source: Paediatr Perinat Epidemiol. 2022 Mar 25;36(5):665–72. doi: 10.1111/ppe.12876 (PMC9543339; doi:10.1111/ppe.12876)
Supplement: Supplementary file 1 — Table S1 [file PPE-36-665-s001.docx]

**eTable 1. Differences in skeletal outcomes between VLBW adults and their term born siblings using linear mixed models.**

|  | **Model 1** | **Model 2** | **Model 3** |
| --- | --- | --- | --- |
|  | Estimate (95% CI) | Estimate (95% CI) | Estimate (95% CI) |
| **Femur** |  |  |  |
| BMD femoral neck Z-score | -0.25 (-0.47, -0.02) | -0.25 (-0.47, -0.02) | -0.14 (-0.39, 0.10) |
| BMC femoral neck (g) | -0.35 (-0.54, -0.16) | -0.34 (-0.53, -0.16) | -0.08 (-0.25, 0.08) |
| Femoral neck area (cm^2^) | -0.15 (-0.25, -0.06) | -0.16 (-0.25, -0.06) | -0.01 (-0.08, 0.06) |
| **Spine** |  |  |  |
| BMD L1-L4 Z-score | -0.06 (-0.31, 0.19) | -0.07 (-0.31, 0.17) | 0.05 (-0.21, 0.31) |
| BMC L1-L4 (g) | -3.60 (-6.37, -0.82) | -3.57 (-6.38, -0.68) | 0.98 (-1.44, 3.41) |
| BMAD L1-L4 (g/cm^2^) | -0.00 (-0.00, 0.00) | -0.00 (-0.00, 0.00) | 0.00 (-0.00, 0.00) |
| **Whole body** |  |  |  |
| BMD whole body Z-score | -0.06 (-0.28, 0.15) | -0.06 (-0.28, 0.15) | 0.01 (-0.24, 0.22) |
| BMC whole body (g) | -236 (-332, -141) | -231 (-326, -136) | -52 (-120, 15) |

Effect size estimates calculated using mixed linear regression models accounting for both preterm birth status and siblingship. A negative value represents a lower result for VLBW subjects compared to term siblings.
Model 1: adjusted for age at clinical examination, sex, and maternal smoking during pregnancy.
Model 2: as model 1 with additional adjustment for BMI measured at clinical examination.
Model 3: as model 2 with additional adjustment for height measured at clinical examination.
BMD = Bone mineral density, g/cm^2^; BMC = Bone mineral content, g; BMAD = Bone mineral apparent density (Bone mineral content L1-L4 divided by area L1-L4^1.5)
